# Genome Wide Identification and Expression Profiling of *SWEET* Genes Family Reveals Its Role During *Plasmodiophora brassicae*-Induced Formation of Clubroot in *Brassica rapa*

**DOI:** 10.3389/fpls.2018.00207

**Published:** 2018-02-28

**Authors:** Hong Li, Xiaonan Li, Yuanhu Xuan, Jing Jiang, Yangdou Wei, Zhongyun Piao

**Affiliations:** ^1^College of Horticulture, Shenyang Agricultural University, Shenyang, China; ^2^College of Plant Protection, Shenyang Agricultural University, Shenyang, China; ^3^Department of Biology, University of Saskatchewan, Saskatoon, SK, Canada

**Keywords:** *Brassica rapa*, *SWEETs*, sugar translocation, *Plasmodiophora brassicae*, clubroot

## Abstract

*Plasmodiophora brassicae* is a soil borne pathogen and the causal agent of clubroot, a devastating disease of *Brassica* crops. The pathogen lives inside roots, and hijacks nutrients from the host plants. It is suggested that clubroot galls created an additional nutrient sink in infected roots. However, the molecular mechanism underlying *P. brassicae* infection and sugar transport is unclear. Here, we analyzed sugar contents in leaves and roots before and after *P. brassicae* infection using a pair of Chinese cabbage near-isogenic lines (NILs), carrying either a clubroot resistant (CR) or susceptible (CS) allele at the *CRb* locus. *P. brassicae* infection caused significant increase of glucose and fructose contents in the root of CS-NIL compared to CR-NIL, suggesting that sugar translocation and *P. brassicae* growth are closely related. Among 32 *B. rapa SWEET* homologs, several *BrSWEETs* belonging to Clade I and III were significantly up-regulated, especially in CS-NIL upon *P. brassicae* infection. Moreover, *Arabidopsis sweet11* mutant exhibited slower gall formation compared to the wild-type plants. Our studies suggest that *P. brassicae* infection probably triggers active sugar translocation between the sugar producing tissues and the clubbed tissues, and the *SWEET* family genes are involved in this process.

## Introduction

*Plasmodiophora brassicae* causes clubroot disease, a most challenging disease of the Brassicaceae family worldwide (Dixon, 2009). *P. brassicae* belongs to an obligate biotrophic protist in the *Plasmodiophorids* within the Rhizaria. The life cycle of *P. brassicae* is divided into three stages: the survival of resting spores in soil, the root hair infection (primary infection), and the cortex infection (secondary infection) (Schwelm et al., 2015; Schuller and Ludwig-Müller, 2016). After infection of the plant roots, *P. brassicae* colonization leads to swelling roots and gall formation, eventually inhibiting roots to uptake nutrients and water from the soil. As one of the most economically serious diseases in *Brassica* crops, clubroot resulted in significant agricultural losses. Moreover, *P. brassicae* can survive in the form of resting spores at least 6–8 years in the soil (Karling, 1968), making it difficult to control by chemicals. Therefore, development of clubroot-resistant (CR) varieties is one of the most economic and effective ways to control clubroot disease. In our previous study, a dominant clubroot resistant gene *CRb*, mapped to the region of 23.667 Mb to 23.752 Mb on *B. rapa* A03 chromosome, has been identified (Piao et al., 2004; Zhang et al., 2014) and successfully introgressed into a Chinese cabbage inbred line (Zhang et al., 2012).

Sugars are the main carbon source for the living organisms including pathogens and their host plants. In plants, sugars are synthesized in the photosynthetic organs and allocated into the sink organs via long-distance transport. Sugars produced in the living plants not only play key roles for plant growth and development, but also are transferred or secreted into the plant surface. Sugars secreted by plants could be used as carbon supply for pathogen growth (Patrick, 1989; Chen et al., 2010). Especially, biotrophic microbes obtain nutrients from host cells by extracting sugars (Manck-Götzenberger and Requena, 2016; Roman and Rathjen, 2017). In terms of *P. brassicae*, it has been reported that 2% sucrose solution could promote germination of resting spores (Guo, 2001). In addition, accumulation of soluble sugars (hexoses and sucrose) was found in the galls of *P. brassicae*-infected *Arabidopsis thaliana* (Evans and Scholes, 1995; Brodmann et al., 2002) and *Brassica* plants (Williams et al., 1968; Keen and Williams, 1969), indicating that the galls might act as a newly established carbon sink during clubroot development. Sugar transporters, including well known gene families such as MSTs (monosaccharide transporters) (Toyofuku et al., 2000), SUTs (sucrose transporters) (Reinders et al., 2008, 2011), and SWEETs (sugar will be eventually exported transporters) (Chen et al., 2012) are responsible for translocate monosaccharides or disaccharides. Recently, transcriptome studies revealed that several genes involved in sugar transport and metabolism strongly up-regulated during gall formation in clubroot-infected *Arabidopsis* (Siemens et al., 2006; Malinowski et al., 2012). A recent transcriptome profiling performed on both shoot and root tissues of *A. thaliana* challenged by *P. brassicae* also pointed that genes involved in sucrose and starch biosynthesis were differently expressed in shoot and root (Irani et al., 2018). These transcriptomic and metabolic experiments provided insights that sugar accumulation is important for gall formation, probably depending on some sugar transport genes. Moreover, during the process of plant-pathogen co-evolution, the pathogens have evolved a mechanism to compete for sugars with infected host cells to sustain their life cycle (Doidy et al., 2012; Lemoine et al., 2013). Competition for sugars at the plant–pathogen interface is likely controlled by the sugar transporters mentioned above (Toyofuku et al., 2000; Doidy et al., 2012), thus regulation of those transporters would give a new insight into disease control.

SWEETs are a class of proteins responsible for transporting sugar across cell membranes, involving in diverse physiological processes of various plant species, such as in reproductive development (Ge et al., 2000; Yang et al., 2006; Guan et al., 2008), leaf senescence (Quirino et al., 1999; Seo et al., 2011), and plant responses to abiotic and biotic stresses in *Arabidopsis* and rice (Chu et al., 2006; Yang et al., 2006; Antony et al., 2010; Chen et al., 2010; Liu et al., 2011). *SWEETs* genes are evolutionally conserved in both prokaryotes and eukaryotes (Gamas et al., 1996; Artero et al., 1998; Chong et al., 2014; Jian et al., 2016), but plant species possess more *SWEETs* than that in animals and prokaryotes (Yuan and Wang, 2013). Phylogenetic analysis of plant *SWEET* genes revealed that they can be grouped into 4 clades as firstly defined in *A. thaliana* (Chen et al., 2010). It is appeared that SWEETs grouped into clades I and II have priority to transport hexoses, while clade III and IV proteins are preferentially transport sucrose and fructose, respectively (Chen et al., 2010, 2012; Lin et al., 2014). Characterization of SWEET proteins indicated that they contain 7 transmembrane (TM) helices carrying two MtN3/saliva domains in plants and animals, while only one MtN3/saliva domain with 3 TM helices exists in SWEET proteins of prokaryotes (Wang et al., 2014). SWEETs localized on the plasma membrane to import or export sugars (Chen et al., 2012). However, SWEET is a distinct transporter family which mediates energy-independent influx and efflux of sugars compared to MSTs and SUTs, both of which are energy-dependent (Reinders et al., 2008, 2011; Yuan and Wang, 2013). Some SWEETs harboring two MtN3/saliva domains functioned as low-affinity glucose and sucrose transporters, such as glucose transporters *AtSWEET4*, *AtSWEET5*, *AtSWEET7*, and *AtSWEET13*, and sucrose transporters *AtSWEET11* and *AtSWEET12* in *Arabidopsis* as well as *OsSWEET11* and *OsSWEET14* in rice (Chen et al., 2010, 2012).

SWEETs are also crucial to regulate carbon transport in parasitism and pathogens interaction. Some members of the *SWEET* genes have been demonstrated as the targets of extracellular pathogens. During the host–microbe interaction, the pathogen infection regulates expression patterns of the *SWEET* genes to facilitate the pathogen obtaining the sugar. The first identified *MtN3* from *Medicago truncatula* was found to be involved in the interaction between the host and *Rhizobium meliloti* (Gamas et al., 1996). Indeed, induction of *SWEET* genes upon pathogen infection has also been reported in *Arabidopsis*, rice, and other plant species. In *Arabidopsis*, at least 9 *SWEET* members (*AtSWEET2*, -*4*, -*7*, -*8*, -*10*, -*11*, -*12*, -*15*, and *-17*) were differentially regulated following infection of different types of pathogens (Ferrari et al., 1994; Chen et al., 2010). In rice, recessive alleles of *Xa13/OsSWEET11*, *Xa25/OsSWEET13*, and *OsSWEET14*, respectively, were identified to be associated with resistance to the rice bacterial blight caused by *Xanthomonas oryzae* pv. *oryzae* (*Xoo*) (Chu et al., 2006; Yang et al., 2006; Antony et al., 2010; Chen et al., 2010; Liu et al., 2011). The dominant alleles of these 3 genes functioned as the low-affinity glucose and sucrose transporters (Chen et al., 2010, 2012), were specifically and transcriptionally activated by the transcription activator-like (TAL) effector after *Xoo* infection (Antony et al., 2010; Yuan et al., 2011). Strong up-regulation of *VvSWEET4* was found in grapevine upon infection with *Botrytis cinerea* (Chong et al., 2014). In the *Arabidopsis*–*P. brassicae* interaction, *P. brassicae* infection strongly induced the expression of *AtSWEET15* during the gall formation (Siemens et al., 2006).

Chinese cabbage belonging to *Brassica rapa* species, is one of the most important leaf vegetables widely cultivated in China, Korea, and Japan, while it is seriously affected by clubroot disease. To better understand the accumulation and transportation of sugars, as well as the role of SWEETs during *P. brassicae*-induced gall formation in Chinese cabbage, we examined the sugar contents in roots and leaves of the Chinese cabbage clubroot resistant (CR) and susceptible (CS) near isogenic lines (NILs) after infection of *P. brassicae*. In addition, the response of *B. rapa SWEET* (hereafter *BrSWEET*) sugar transporters to *P. brassicae* infection as well as clubroot disease development in *Arabidopsis sweet11* mutant was examined.

## Materials and Methods

### Identification of *SWEET* Family Genes in *B. rapa*

To identify the *SWEET* genes in *B. rapa* genome, amino acid sequences of 17 *A. thaliana SWEET* (*AtSWEET*) genes were used as the bait to search against the *B. rapa* database (BRAD^1^) by performing a BLASTP analysis. The physicochemical parameters, including molecular weight (kDa) and isoelectric points (pI), were calculated according to the pI/Mw tool at online ExPASy^2^ database. Proteins containing MtN3/saliva domain, the typical transmembrane domains of eukaryotic SWEET, were defined to belong to the *B. rapa* SWEET gene family (BrSWEETs). *BrSWEETs* paralogous genes to the *AtSWEET* was named by adding a suffix (a, b, c…) according to E values from high to low, representing sequence similarity level to the corresponding AtSWEET.

### Multiple Sequence Alignment and Phylogenetic Analysis of *BrSWEET* Family Genes

To better understand the evolutionary relationships among *A. thaliana*, *B. napus*, and *B. rapa* SWEETs gene family, multiple sequence alignment of all BrSWEET sequences was conducted using the ClustalX program. An unrooted phylogenetic analysis was performed using the neighbor-joining (NJ) method of the MEGA5 program using the full-length SWEET amino acid sequences of *A. thaliana*, *B. napus*, and *B. rapa*. A bootstrap test was performed with 1000 replicates.

### Chromosomal Location and Gene Structure of *BrSWEET*s

All identified SWEET genes were mapped to *B. rapa* chromosomes using Mapchart (Version 2.1) software based on their physical position available at the *B. rapa* genome database^1^. Gene Structure Display Server (GSDS)^3^ was used to draw the gene structure diagram of the *BrSWEET* family genes according to the genomic sequences and the corresponding coding sequence of each *BrSWEET* gene. To further confirm the coding sequence, all *BrSWEETs* were queried against on line EST database deposited in NCBI and sequences generated from our lab RNA-seq data of *B. rapa* (Chen et al., 2016) with BLAST.

### Analysis of the Protein Structure and the Conserved Motifs of *BrSWEET* Family

TMHMM Server 2.0^4^ was utilized to forecast the protein structure of BrSWEET family. We performed the MEME (Multiple Expectation maximization for Motif Elicitation) analysis on the predicted BrSWEET with the conditions: (1) optimum motif width was set to 6 and 50; (2) maximum number of motifs was designed to 10 motifs; (3) the iterative cycles were set by default.

### Plant Growth and *P. brassicae* Infection

A pair of Chinese cabbage near-isogenic lines (NIL), carrying either the CR or clubroot-susceptible (CS) allele at the *CRb* locus, were used in our study. Briefly, CR-NIL was bred by introgressing *CRb* gene from clubroot-resistant line ‘CR Shinki DH’ to the susceptible inbred line “BJN3-2” based on marker-assisted selection (Zhang et al., 2012). All the plants were maintained in the culture room under a 16 h photoperiod at 25°C. The single-spore isolate (Pb4) of *P. brassicae* was propagated in CS Chinese cabbage. Resting spores were isolated from homogenized clubbed roots and diluted to a density of 10^7^/mL with sterile distilled water until inoculation. Thirteen-day-old seedlings of CR and CS NILs were inoculated by injecting 1 mL of resting spore suspension into the soil around each plant (Zhang et al., 2012). Plants supplemented with the same volume of water were used as the control. Leaves, roots and hypocotyls from 10 individuals were collected at 0, 0.5, 1, 1.5, 2, 3, 4, 6, and 9 days post inoculation (dpi) for gene expression analysis, respectively. Also, sugar contents of the leaves and roots were determined. To verify the successful infection, a pair of NILs was maintained at the culture room for 30 days. Three biological replicates were included for each treatment, and each replicate contained 10 plants.

For analyzing clubroot formation in *Arabidopsis*, T-DNA insertion mutant *sweet11* (SALK_073269.20.35.X) from the Salk collection and wild type Col-0 were grown for 3 weeks in a chamber with 12 h light/12 h dark cycle. The phenotype of *Atsweet11*, including plant height, leaf size, leaf color, and flowering showed no difference to wild type Col-0. *P. brassicae* was infected and the gall formation was checked after 3, 4, and 5 weeks. Three biological replicates were conducted for each treatment, and each replication contained 10 plants. The disease rate (DR) and the disease index (DI) was evaluated after inoculation. Disease symptoms were scored as follows: 0, no symptoms; 1, a few small clubs on the lateral roots; 2, larger clubs on the lateral roots; 3, swelling of the main roots; 4, severe galling of tissues of both lateral and main roots. The DI was calculated according to the formula DI = [nw] × 100/4T, where n is the number of plants in each class, w is disease symptoms (0–4), and T is the total number of plants tested (Chaube and Singh, 1991; Siemens et al., 2010). Data on the phenotype of disease index of the tested lines were analyzed for statistical significance using analysis of variance (ANOVA) conducted using SPSS 17.0.

### Total DNA, RNA Extraction and the Quantitative Real-Time PCR Analysis

For pathogen DNA quantification, total DNA was extracted from 1 g roots of the *Arabidopsis sweet11* mutants and wild type Col-0 by DNAsecure plant kit (Tiangen, Beijing, China), according to the manufacturer’s instructions. DNA quantity was estimated with Nanodrop instrument and final concentration was adjusted to 10 ng/μl for each PCR reaction. The *Arabidopsis* F-box protein gene mentioned by Lemarie et al. (2015) was used for normalization and the *P. brassicae* target gene was described by Faggian et al. (1999). Quantitative real-time PCR reaction was performed following by Lemarie et al. (2015). The relative DNA quantities were expressed as the ratio between the DNA quantities of *P. brassicae* and the plant DNA value. Three replications were performed for each reaction. Significant differences between the mean values among *Arabidopsis* wild type and mutants were determined according to ANOVA analysis at *P* < 0.01 using SPSS 17.0.

For SWEET gene expression analysis, total RNA was extracted from the roots, hypocotyls and leaves of CR and CS NILs by using the RNA prep Pure Plant Total RNA Extraction Kit (Tiangen, Beijing, China) according to the manufacturer’s instructions. cDNA was synthesized from 1 μg RNA after removement of genomic DNA. The quantitative real-time PCR (qRT-PCR) analysis was performed as described by Jain et al. (2006). Briefly, the cDNA samples were used as template and mixed with 200 nmol of each primer and SYBR Green PCR Real Master Mix (Tiangen, Beijing, China) for real-time PCR analysis using ABI 7500 Real Time PCR System and Software 7500 ver. 2.0.3 (Applied Biosystems, United States) according to the manufacturer’s instructions. The thermal cycling was as following: 95°C for 3 min; 40 cycles of 95°C for 30 s, 57°C for 30 s, and 68°C for 1 min. The fluorescence signal was collected during the elongation at 68°C of every cycle. The gene specific primer sequences were listed in **Supplementary Table S2**. The relative expression levels for each of the *SWEET* genes were quantified with respect to the internal standard, *Actin* and *18sRNA*. The experiments were repeated at least three times.

### Soluble Sugars Extraction and Content Determination

Soluble sugars were extracted from the roots and leaves of CR and CS NILs. After grinding the tissues, 1 g of powder samples were dissolved in 80% ethanol and were kept at 80°C for 1 h. The extracts were concentrated and dissolved with 1 ml deionized and distilled H_2_O. Sucrose, glucose and fructose were assayed by high performance liquid chromatography (HPLC) using a NH_2_ Analytical HPLC column (Waters, Milford, MA, United States) with 75% acetonitrile and 25% double-distilled H_2_O as solvent, the velocity of 1.0 ml⋅min^-1^ and refractometer Parallax detection, according to manufacturer’s directions (Siemens et al., 2010). Soluble sugars were extracted from three biological replicates at each time point as aforementioned treatments. HPLC analysis were repeated three times for each treatment.

## Results

### The Variation of Soluble Sugar Contents After *P. brassicae* Infection

**FIGURE 1:**
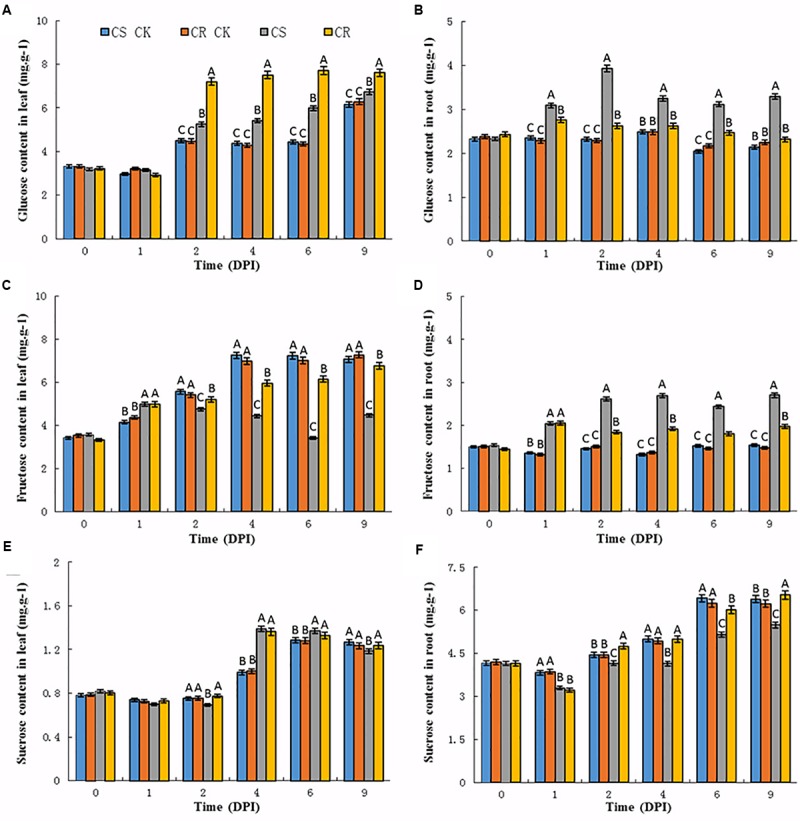
Sugar contents in the leaves and roots of Chinese cabbage after *P. brassicae* infection. **(A)** Glucose, **(C)** fructose, **(E)** sucrose contents in Chinese cabbage leaves after *P. brassicae* infection, respectively. **(B)** Glucose, **(D)** fructose, **(F)** sucrose contents in Chinese cabbage roots after *P. brassicae* infection, respectively. Significant differences at least *P* < 0.01 are indicated by different letters. CS CK and CR CK represent the corresponding sugar content in CS-NIL and CR-NIL without *P. brassicae* inoculation. CS and CR represent the sugar content in CS-NIL and CR-NIL after *P. brassicae* inoculation, respectively. Horizontal axis is time point from 0 to 9 days after inoculation (DPI).

To test the effects of *P. brassicae* infection on sugar translocation, the contents of soluble sugars (glucose, fructose, and sucrose) in the leaves and roots of CS and CR NILs were examined before and after infection. The contents of sugars had no significant differences between CS and CR NILs without *P. brassicae* inoculation, but showed variance after infection (**Figure 1**).

Upon *P. brassicae* inoculation, an increase of glucose contents was observed in the leaves and roots of both two genotypes (**Figures 1A,B**). The contents of glucose were slightly increased in the leaves of CS and CR NILs at 1 dpi, while increased from 2 dpi. Compared to the CS-NIL, CR-NIL showed dramatically high content of glucose in the leaves from 2 to 9 dpi. However, the contents of glucose were dramatically increased from 12 to 49% in the roots of the CS-NIL from 1 to 9 dpi, and much less increase of the glucose contents occurred in the roots of the CR-NIL (**Figures 1A,B**).

In the leaves of un-inoculated plants, fructose contents gradually increased in the leaves of both CS and CR NILs at 1 dpi (**Figure 1C**). Generally, the fructose contents were lower in the leaves of both CS-NIL and CR-NIL than that of un-inoculated plants at each time point after *P. brassicae* inoculation. It was noticed that significant decreases of fructose contents were observed in the leaves of CS-NIL compared to CR-NIL from 2 dpi. However, significant increases were observed in the roots of both NILs during the infection time course, especially in CS-NIL. The fructose contents in roots of CS-NIL were higher about 35–42% than that in CR-NIL from 1 to 9 dpi (**Figure 1D**). It could be observed that patterns of fructose and glucose accumulations in the roots of both CS and CR-NILs are similar.

Sucrose contents showed no changes in leaf or root tissues between un-inoculated CS and CR NILs (**Figures 1E,F**). After *P. brassicae* infection, lower sucrose contents were detected at the early infection time points (2 dpi) in the leaves of the CS-NIL than that of the CR-NIL. Thereafter, significant increases of sucrose contents were observed in the leaves of both CS and CR NILs, but no significant differences between two NILs. In the root tissues, sucrose contents showed an increase tendency in inoculated and un-inoculated CR-NILs at the late infection time points (from 4 to 9 dpi). However, much less increases of sucrose contents were detected in the roots of the CS-NIL than that of the CR-NIL from 2 dpi.

In summary, glucose and fructose levels reduced in the leaves of CS-NIL, but increased in the roots after *P. brassicae* infection. Also, sucrose content of CS-NIL was lower than CR-NIL in roots. These results imply that *P. brassicae* infection may activate translocation of sugar from the leaves to roots.

### Identification of *SWEET* Genes in *B. rapa*

To analyze the possible sugar transporter involved in long distance transport, we firstly identified *SWEET* genes in *B. rapa* genome. Based on the similarities with the 17 *Arabidopsis* AtSWEETs, totally 32 SWEETs were identified in *B. rapa*, and named BrSWEET1a to BrSWEET17b on the base of their identity to AtSWEETs (**Supplementary Table S1**). 28 *BrSWEET* genes are multicopy either duplication or triplication of corresponding *AtSWEET*, except for four single copy genes *(BrSWEET8*, *-9*, *-10*, and *-13*). In addition, the orthologous gene of *AtSWEET6* was not found in the *B. rapa* genomes. Majority of *BrSWEET* genes were about 1500 base pairs (bp), and amino acids residues are from 178 to 316. Genes longer than 7.5 kb were also found in *BrSWEET15b* and *BrSWEET17b*. BrSWEETs contained 3–7 TM helices (**Supplementary Figure S1**) harboring one or two conservative MtN3/saliva domains (**Supplementary Figure S2**). The isoelectric points (pI) value of each BrSWEETs was higher than 7.50. Among them, pI of 19 BrSWEETs was higher than 9.0, and others were ranged between 7.59 and 8.97. Noticeably, no single *SWEET* gene was observed to be closely associated with the *CRb* locus (Zhang et al., 2014) on the *B. rapa* genome.

### Phylogenetic Analyses of the *BrSWEET* Genes

**FIGURE 2:**
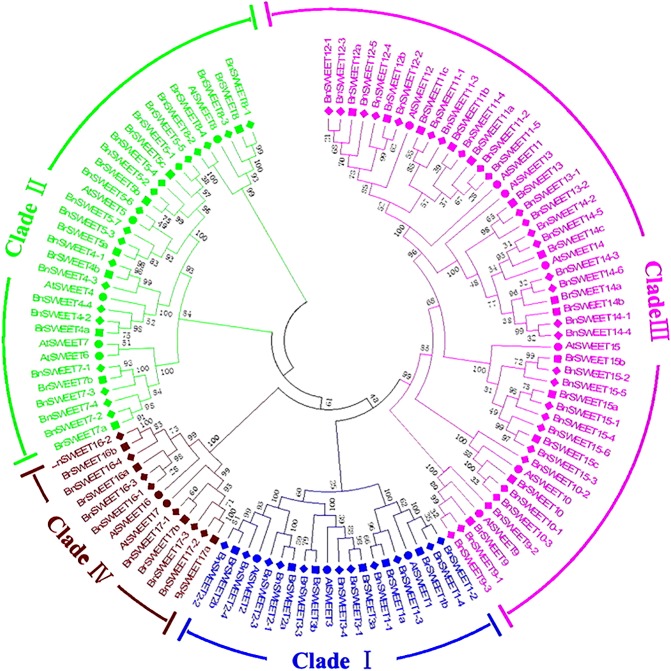
Phylogenetic tree of SWEETs from *B. rapa*, *B. napus*, and *A. thaliana*. The phylogenetic tree was built using the neighbor-joining (NJ) method. The unrooted tree was generated using ClustalW in MEGA5 via SWEETs amino acid sequences from *A. thaliana* (*AtSWEET*), *B. rapa* (*BrSWEET*), and *B. napus* (*BnSWEET*). The roman numerals (I – IV) labeled with various colors indicate different clades. The numbers at the nodes represent bootstrap percentage values based on 1000 replications. Genes from each species are marked with different bullet point colors.

Further, to better understand the evolutional relationships of BrSWEETs, amino acid sequences of the 117 SWEET proteins (17, 32, 68 from *A. thaliana*, *B. rapa*, and *B. napus*, respectively) were aligned and an unrooted phylogenetic tree was constructed (**Figure 2**). All 32 BrSWEET family members contain two conserved regions, and they might have evolved to a closer relationship during evolution (**Supplementary Figure S2**). Seven sister pairs of genes were identified in the phylogenetic trees with very strong bootstrap support (100%) (**Figure 2**). Most of the gene pairs had short branch lengths, suggesting their recent divergence (**Figure 2**).

All of 32 *BrSWEET* genes, together with other 85 members were grouped into 4 major clades, namely clade I, II, III, and IV (**Figure 2**). Gene families in clade III had relative higher molecular weight than others (**Supplementary Table S1**). All BrSWEET and BnSWEET were clustered closely with their AtSWEET orthologs.

### Chromosomal Position of the *BrSWEET* Genes

**FIGURE 3:**
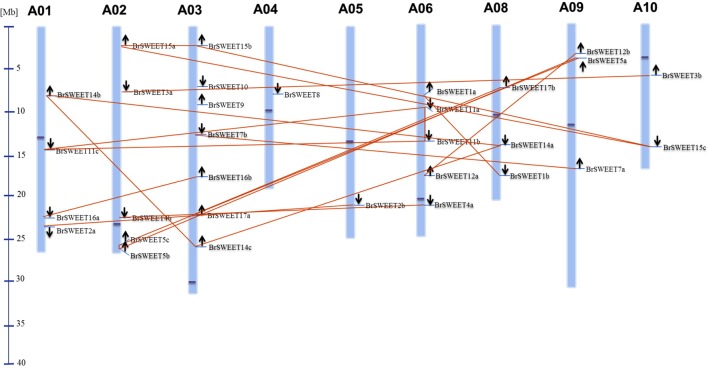
Distribution of *BrSWEET* genes on *B. rapa* chromosomes. Out of 32 Br SWEET genes identified, 31 genes were mapped to the 9 out of 10 chromosomes, and SWEET13 gene is unable to map to the chromosome. Light blue lines represent the gene position on the chromosome. Paralogous genes are connected with lines in orange color. Black arrows indicate the direction of genes. Blue line in the left panel represents the scale of chromosome. Dark blue dot on each chromosome represents the centromere position.

According to the gene loci information, all *BrSWEET* genes were assigned to the 9 chromosomes of *B. rapa* with the exception for *BrSWEET13* (**Figure 3** and **Supplementary Table S1**). Duplication event of *AtSWEET* genes was found in *B. rapa* genomes. 8 *AtSWEET* genes (*AtSWEET1*, -*2*, -*3*, -*4*, -*7*, -*12*, -16, and -*17*) were duplicated and 4 genes (*AtSWEET5*, -*11*, -*14*, and -*15*) are triplicated in *B. rapa*. Most of *BrSWEET* paralogous genes were dispersed to different chromosomes. However, tandem array of 2 *BrSWEET5* and 2 *BrSWEET11* was found on the chromosome A02 and A06, respectively. Other 3 *BrSWEET* genes (*BrSWEET14, -16* and -*17*) were found to be involved in the duplication of chromosomal fragments.

### Gene Structure of *BrSWEET*s

**FIGURE 4:**
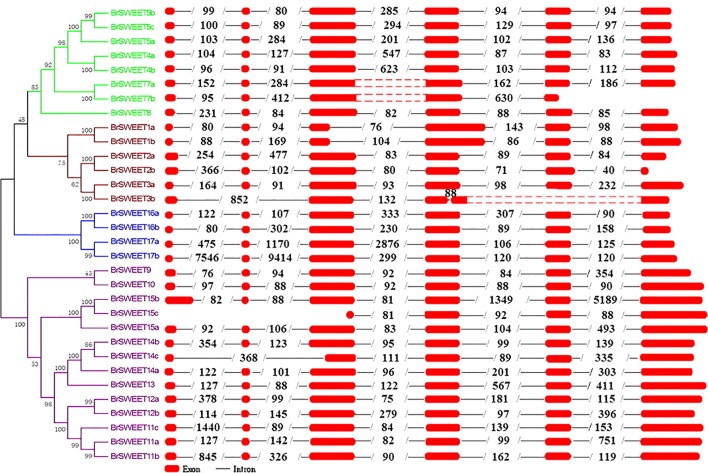
Genomic structures of 32 SWEET genes identified in *B. rapa*. Exon–intron structure of the BrSWEET genes were graphically shown. Exons are represented by red boxes, while introns are represented by gray lines. The boxes with red dot means no sequences in this region, and two red boxes are both sides constitute one exon. The numbers within intron indicate the length of introns.

Exon-intron organizations for each *BrSWEET* gene were analyzed by alignment of full-length cDNA sequences to the genomic DNA sequences of *B. rapa*. Characterization of the gene structure diversity revealed that most *BrSWEET* genes contained 6 exons (**Figure 4**). Five *BrSWEET* genes (*BrSWEET3b*, -*7a*, -*7b*, -*14c*, and -*15c*) appeared to have exon-intron loss variations, probably due to the fact that loss/gain of one or two intron/exon were happened during the evolution of *B. rapa*. Most genes had similar exon lengths, while the length of intron varied obviously for each gene. Among which, *BrSWEET11c*, -*15b*, -17a, and -*17b*, had extremely long intron.

### Conserved Domains and Motifs of BrSWEET Proteins

The common domains for cytosolic localization, apoplastic localization, and transmembrane anchorage were found in all *BrSWEET* genes (**Supplementary Figure S1**), indicating that *BrSWEET* genes are composited with membrane spanning, intracellular and extracellular regions, and responsible for sugar transport. Most of BrSWEETs had 2 conservative MtN3/saliva domains with 7 TM helices (**Supplementary Figures S1**, **S2**), the typical domain of SWEET in eukaryote. However, genes (*BrSWEET3b*, *7b*, and *15c*) with 3 or 4 TM helices were found to have only one complete MtN3/saliva domain.

To investigate the structural diversity further, all *BrSWEET* genes were submitted to MEME program for the conserved motifs structure analysis. As the results, 10 conserved motifs were identified, among which 6 motifs (motifs 1–6) were annotated as MtN3/saliva domains of the SWEET genes (**Supplementary Figure S3**). All genes contained motif 1, 2, 5, and 6 in the C terminal of BrSWEETs However, the order of motif arranged in the N terminal was different. Motif 1-4/5-6-7 was found in clade I, II, and IV, but motif 7-2-3-5-6 in clade III. Additionally, loss of some motifs was observed in a few genes, including motif 1-2-5 in the C terminal of BrSWEET3b and BrSWEET7b, and motif 3-5-6 in the N terminal of BrSWEET15c. Gene families in clade I and II contained a specific motif 4 except for 3 genes in clade II. Motifs 8 and 10 were specific to clade III. The variation of the structural motifs among BrSWEET family suggested functional diversity of the *SWEET* genes.

Two conservative structures of MtN3/saliva domain with TM1 (motif1/2/3), TM2 (motif4/5) and TM3 (motif6), and TM5 (motif1), TM6 (motif2/5) and TM7 (motif6), respectively, were present in all genes except for BrSWEET3b, BrSWEET77b, and BrSWEET15c. These 2 conservative structure domains were consisted of about 85 amino acids, and were located in the similar position (**Supplementary Table S1** and **Supplementary Figure S3**). TM4 is structured with motif 7 or motif 8, and it plays the role of contact between two MtN3/saliva domains.

### Tissue Specific Expression of *BrSWEET* Genes in *B. rapa*

**FIGURE 5:**
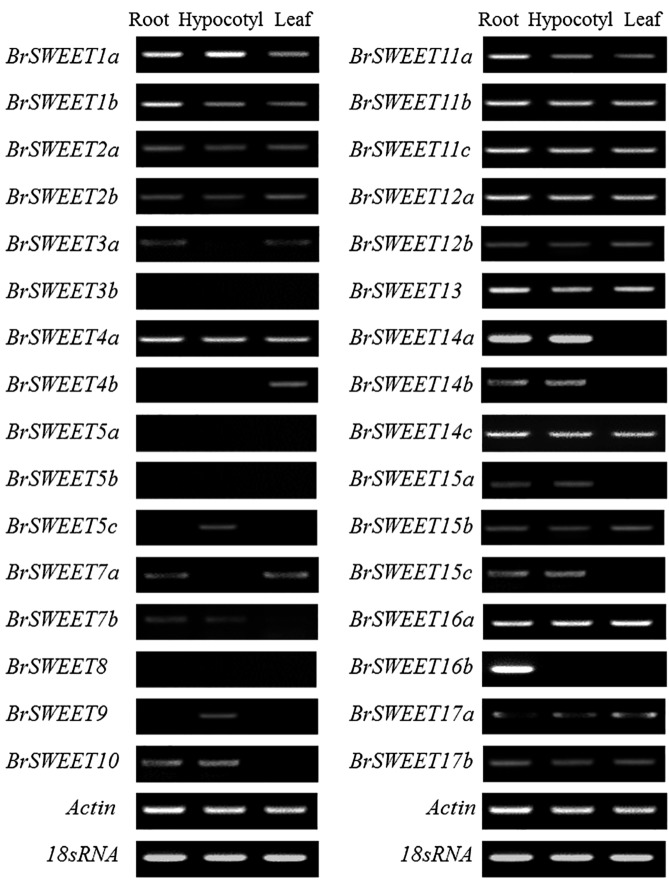
Expression patterns of the SWEET genes in different tissues of Chinese cabbage. Tissue specific expressions of *BrSWEET* genes in *B. rapa* (CS–NIL) were analyzed by semi-quantitative reverse transcription polymerase chain reaction (RT-PCR). Actin and 18srRNA genes were used as the internal control.

To investigate expression patterns of *BrSWEET* genes in *B. rapa*, semi-quantitative RT-PCR analysis was carried out in leaf, hypocotyl and root tissues of uninfected plants of CS-NIL. The semi qRT-PCR data revealed that the transcripts were detected for the majority of *BrSWEET* genes among all tissues (**Figure 5**). Half of the genes were expressed in all tissues. Transcript of four genes, including three duplicated genes (*BrSWEET3b*, -*5a* and -*5b*) and one single copy genes (*BrSWEET8*) were undetectable in any given tissues tested. High transcript abundance of six genes (*BrSWEET7b*, -*10*, -*14a*, -*14b*, -*15a*, and -*15c*) were expressed in the roots and hypocotyls, and two genes (*BrSWEET3a* and *BrSWEET7a*) were predominantly expressed in the roots and leaves. Several genes were expressed in only one kind of tissue, including *BrSWEET4b* in leaves, *BrSWEET5c* and *BrSWEET9* in hypocotyls, and *BrSWEET16b* in roots. The expression profiling of *BrSWEET* genes suggests their possible involvements in broad biological functions during vegetative growth and development of *B. rapa.*

### Expression Profiling of *BrSWEET* Genes in Response to *P. brassicae* Infection

**FIGURE 6:**
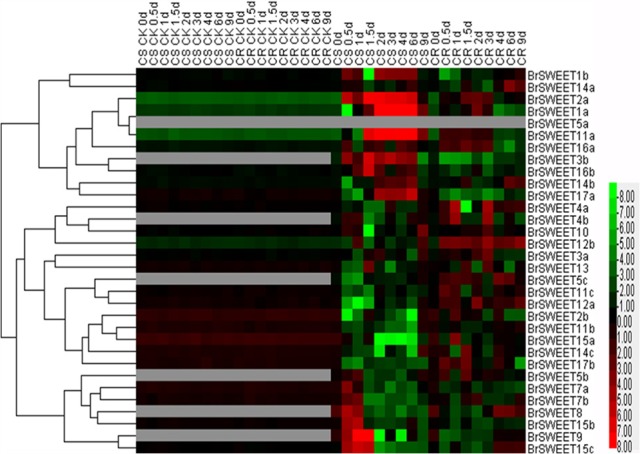
*Plasmodiophora brassicae*-dependent SWEET gene expressions in the root of Chinese cabbage. Hierarchical clustering and heatmap representation show time course responses of SWEET gene expressions in the root of Chinese cabbage after *P. brassicae* infection. The expression levels of genes are presented using fold-change values transformed to Log2 format. The data obtained by quantitative RT-PCR correspond to the levels of SWEETs in total RNA samples extracted from roots before and after infection of *P. brassicae*. The data indicate the relative expression levels normalized to that of the internal control Actin or 18srRNA. Gray color means no signal detected,while red and green colors correspond to up- and down-regulations of the SWEET gene expressions, respectively.

**FIGURE 7:**
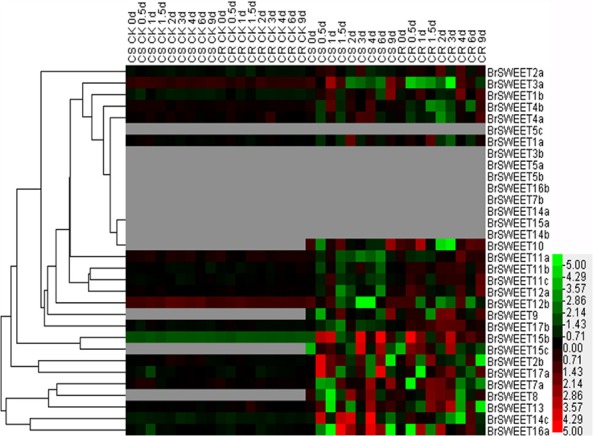
Expression patterns of the SWEET genes in the leaves of Chinese cabbage after infection of *P. brassicae*. Hierarchical clustering and heatmap representation show time course responses of SWEET gene expressions in the leaves of Chinese cabbage after *P. brassicae* infection. The expression levels of genes are presented using fold-change values transformed to Log2 format. The data obtained by quantitative RT-PCR correspond to the levels of SWEETs in total RNA samples extracted from leaves before and after infection of *P. Brassicae*. The data indicate the relative expression levels normalized to that of the internal control Actin or 18srRNA. Gray color means no signal detected, while red and green colors correspond to up- and down-regulation of the SWEET gene expressions, respectively.

To study the expressions of *BrSWEET* gene responsive to the *P. brassicae* infection, the expression patterns were determined among 3 different tissues (leaf, hypocotyl, and root) at different time points post *P. brassicae* inoculation by qRT-PCR. The heatmap results revealed that the expression patterns among *BrSWEET* genes were variable depending on the tissues and treatments (**Figures 6**, **7** and **Supplementary Figure S4**).

*BrSWEETs* expressed differentially in the roots of CS and CR NILs after infection of *P. brassicae* (**Figure 6**). In comparison with un-inoculated plants, among all *BrSWEET* genes 14 (*BrSWEET1a*, *-1b*, -*2a*, -*3b*, -*7a*, *-7b*, -*11a*, -*14a*, -*14b*, -*15b*, -*15c*, -*16a*, -*16b*, and -*17a*) and 15 (*BrSWEET1a*, *-2a*, -*3a*, -*4a*, -*10*, -*11a*, -*11c*, -*12a*, -*12b*, -*14a*, -*14b*, -*14c*, -*15c*, -*16a*, and -*17b*) were up-regulated in CS-NIL and CR-NIL respectively. Transcripts of other genes were undetectable or down-regulated during *P. brassicae* infection. *BrSWEET3b, -4b*, *-5b*, *-5c*, *-8*, and -*9* were not expressed in un-inoculated plants, but were induced expression in the *P. brassicae*-infected roots of both CS and CR NILs. Among them, *BrSWEET3b*, -*8* and -*9* were strongly induced in CS-NIL and *BrSWEET4b*, -*5b*, and -*5c* were significantly up-regulated in CR-NIL. By comparing between CS and CR NILs after *P. brassicae* infection, expression levels of 15 genes (*BrSWEET1a*, -*1b*, -*2a*, *-3b*, *-7a*, *-7b*, -*8*, *-9*, -*11a*, *-14b*, *-15b*, *-15c*, *-16a*, *-16b*, and -*17a*) were higher in the CS-NIL, but expression levels of other 16 genes were relatively higher in the CR-NIL. Most notably, the expression of *BrSWEET1a*, -*2a*, -*9*, -*11a*, and -*15c* was strongly up-regulated (over 1000-folds) in the roots of CS-NIL, and the expression of *BrSWEET4a, -5c*, -*12a*, and -*12b* was significantly up-regulated in the roots of CR-NIL (**Figure 6**). Clustering and heatmap analysis further revealed that among up-regulated *BrSWEET* genes in CS-NIL, several genes (*BrSWEET7a*, *-7b -8*, *-9*, *-15b*, and *-15c*) showed transiently induced expression at the early infection time points (0.5–1.5 dpi), whereas induced expressions of other genes (*BrSWEET1a*, -*1b*, -*2a*, *-3b*, -*11a*, *-16b*, and -*17a*) maintained up to the late infection time points at 6–9 dpi (**Figure 6**).

In the hypocotyls, expressions of *BrSWEET3a*, -*3b*, -*4b*, -*5b*, -*7a*, -*8*, and -*16b* were strikingly induced on both CR and CS-NILs by the infection of *P. brassicae*. Eleven genes (*BrSWEET4a*, -*5b*, -*8*, -*10*, -*11a*, -*11b*, -*12b*, -*14c*, -*15a*, -*17a*, and -*17b*) exhibited down-regulation, and other 20 genes were up-regulated in the hypocotyls of CS and CR NILs after infection of *P. brassicae*. Remarkably, 2 *SWEET* genes (*BrSWEET9* and -*16a*) were found to be significantly up-regulated specifically in hypocotyls of CS-NIL after infection of *P. brassicae* (**Supplementary Figure S4**).

In leaf tissues, 9 *BrSWEET* genes (*BrSWEET3b*, -*5a*, -*5b*, -*5c*, -*7b*, -*14a*, -*14b*, -*15a*, and -*16b*) showed undetectable levels of expression on both CR and CS NILs before or after *P. brassicae* infection, while 4 genes (*BrSWEET8*, -*9*, -*10*, and -*15c*) showed enhanced expressions on both CR and CS NILs upon *P. brassicae* infection. Among these four *P. brassicae*-induced genes, *BrSWEET9* had higher expression levels in CR-NILs than CS-NILs, whereas *BrSWEET8*, *BrSWEET10* and *BrSWEET15c* showed similar expression patterns between CR and CS NILs. Most of the expressed genes either in CR or CS NILs showed differential regulations at different infection time points. Six *SWEET* genes (*BrSWEET2b*, -*14c*, -*15b*, *-15c*, -*16a*, and -*17a*) were found to be the most significantly up-regulated in the leaves of CS-NIL (**Figure 7**).

### Pathogenicity Assays on *Arabidopsis AtSWEET* Mutant

**FIGURE 8:**
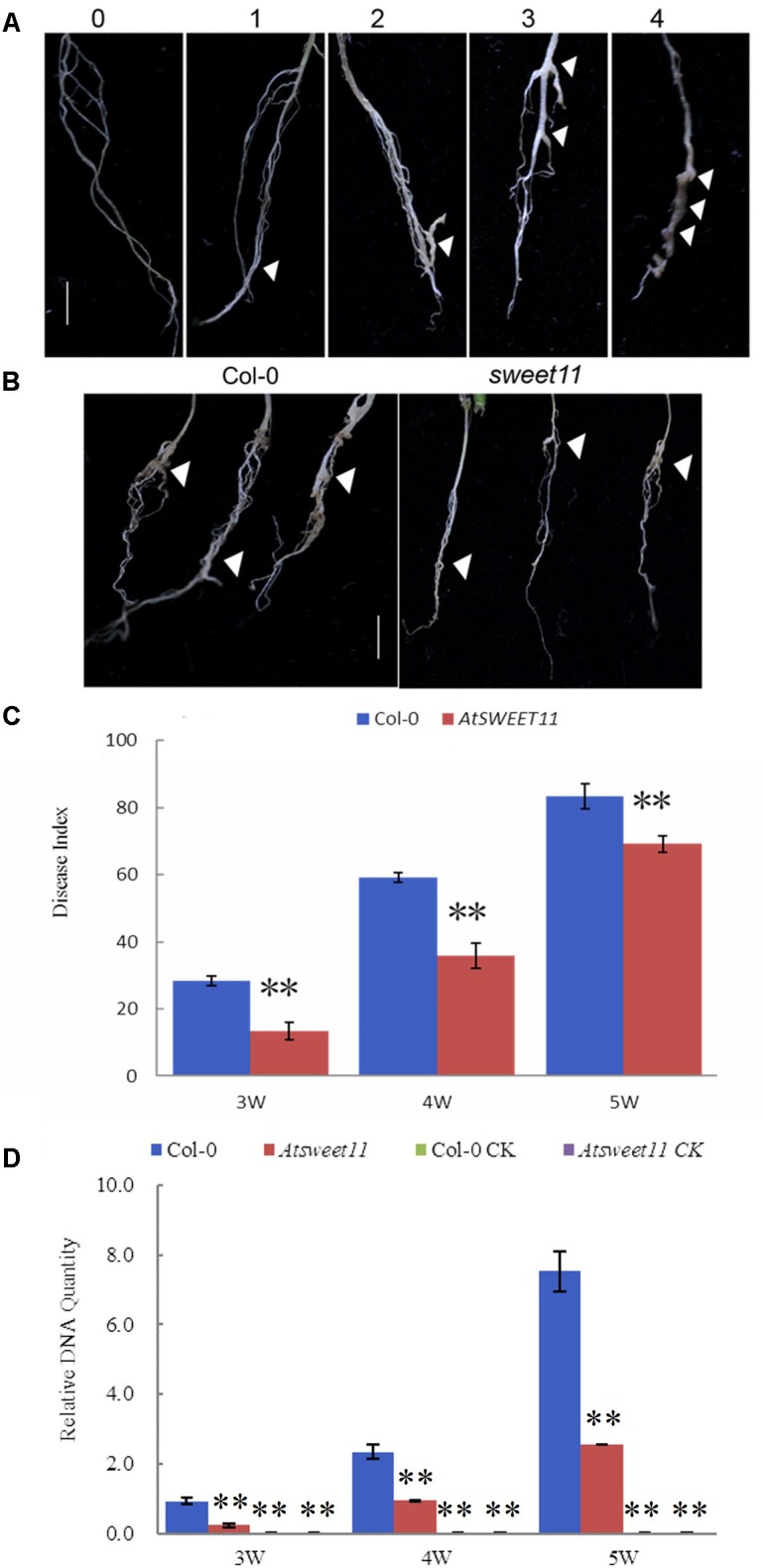
The disease index (DI) of clubroot development in *A. thaliana* after inoculation with *P. brassicae*. **(A)** Disease symptoms were scored as the follows: 0, no symptoms; 1, a few small clubs on the lateral roots; 2, larger clubs on the lateral roots; 3, swelling of the main roots; 4, severe galling of tissues of both lateral and main roots. **(B)** The root morphology of the sweet11 mutant (*sweet11*) and the corresponding wild-type plants (Col-0) after infection of *P. brassicae*. Arrows point to the position of gall formation. **(C)** The disease index (DI) was calculated according to the formula DI = [nw] × 100/4T, where n is the number of plants in each class, w is disease symptoms (0–4), and T is the total number of plants tested. Data were collected during an infection time course at 3, 4, and 5 weeks post inoculation. Significant differences between the wild-type (Col-0) and the sweet11 mutant are indicated by asterisks (*P* < 0.01). **(D)** The relative pathogen DNA content of the sweet11 mutant (*sweet11*) and the corresponding wild-type plants (Col-0) after infection of *P. brassicae*. Horizontal axis is time point after *P. brassicae* inoculation. Vertical axis is the relative DNA quantities. Col-0 CK and *Atsweet11* CK represent the relative DNA quantities of *Arabidopsis* wild type and mutant without *P. brassicae* infection as control.

*AtSWEET11* in *Arabidopsis* mediates sucrose efflux in phloem parenchyma cells, promoting sucrose loading into the phloem by SUC2 for long distance transport (Chen et al., 2012). Our results revealed that *P. brassicae*-infected plants showed reduced sucrose contents in the leaves and roots, while increased glucose and fructose contents in the roots of *B. rapa*. Moreover, increased expression of *BrSWEET11a* gene occurred in root tissues upon *P. brassicae* infection. These results suggest that *SWEET11* gene might be involved in host sugar transport into diseased clubroot tissues. To investigate possible involvement of *SWEET11* during *P. brassicae* pathogenesis, *Arabidopsis sweet11* mutant (*sweet11*) and its corresponding wild type Col-0 line were inoculated with *P. brassicae.* Disease development and gall formation were examined at 3, 4, and 5 weeks post inoculation (wpi) with *P. brassicae* resting spores. At 3 and 4 wpi, *sweet11* mutants showed significantly lower disease incidence than that of the wild-type plants (**Figure 8**). After 5 wpi, all plants including mutant and the wild-type produced disease symptoms with various levels of disease severities. The disease index (DI) value of *sweet11* mutants was significantly lower than that of the wild-type at all time points post inoculation (**Figure 8C** and **Supplementary Table S3**). Furthermore, we detected the relative pathogen DNA quantities of both wild type and sweet11 mutants after inoculation. The results showed that the *P. brassicae* content was continuously increased in both *sweet11* mutants and Col-0 of *Arabidopsis* from 3 to 5 wpi (**Figure 8D**). Meanwhile, the DNA content of *P. brassicae* in the *sweet11* mutants was significantly lower than wild type Col-0 at each time point (**Figure 8D** and **Supplementary Table S3**). All the results indicated that the progression of clubroot development in the *sweet11* mutant was significantly delayed when compared to that of the wild-type plants. Thus, *Arabidopsis SWEET11* gene is suggested to be responsive to the *P. brassicae* infection, and contributes to host susceptibility to the disease.

## Discussion

The plant pathogens have a tendency to change the sugar transport and distribution in host tissues which has been reported in wheat and rice (Sutton et al., 1999; Yuan and Wang, 2013), despite different types of sugars involved. It was proposed that enhanced sugar efflux from host cells could lead to sucrose and hexose accumulation in the apoplast, which are subsequently taken up by the sugar transporters of the fungal pathogens (Doidy et al., 2012). In this study, we observed that the contents of glucose and fructose were significantly increased in the clubroot tissues of CS–NIL than that of CR-NIL, but decreased in the leaves after infection with *P. brassicae*. The similar results were also observed in *P. brassicae*-infected *A. thaliana*, in which glucose and fructose accumulated highly in galls and decreased in the leaves (Brodmann et al., 2002). Translocation of ^14^C-labeled photosynthates from the leaves to the galls/roots of infected *B. rapa* plants was also reported (Evans and Scholes, 1995). All these studies indicate that altered sugar transport and redistributions from aboveground leaves to infected roots underlies the clubroot disease development.

The *SWEET* genes are prevalent and evolutionally conserved gene families in higher eukaryotes. In plants, *SWEET* genes not only play key roles for the sugar transport from the source to the sink organs during plant growth and development (Chen et al., 2012; Patil et al., 2015), but also are involved in the sugar and energy translocation during plant–microbe interactions. In this study, a total of 32 *BrSWEET* genes corresponding to 16 *AtSWEET* family genes were identified in the *B. rapa* genome. Expression profiling indicated that many of these *SWEET* genes were differentially expressed in the roots and leaves of *B. rapa* plants upon *P. brassicae* infection. Furthermore, the distinct soluble sugar contents occurred in the roots and leaves between the clubroot resistant and susceptible genotypes after *P. brassicae* infection. These results suggest that some of the *BrSWEET* genes are likely involved in the *B. rapa*–*P. brassicae* interaction.

### Structure and Evolution of the *BrSWEET* Genes

The number of *SWEET* genes is largely variable among green plant species. For example, unicellular and green algae have only 1–3 copies of *SWEET* genes, but 18–23 genes in monocots, and 15–68 genes in dicots (Chong et al., 2014; Patil et al., 2015; Jian et al., 2016). *Arabidopsis*, the closely related species to *Brassica* genus, contains 17 *SWEET* genes (Kühn et al., 2010). In consistence with the genome triplication of *Brassica* species since its divergence from *Arabidopsis* (Wang et al., 2011), much higher numbers of *SWEET* genes were identified in *B. rapa*. These suggest that there may be functional redundancy or divarication between the respective SWEET members. However, the occurrence of gene loss during polyploid speciation was also found in the *B. rapa* genome corresponding to 5 *Arabidopsis AtSWEET* genes, i.e., the *AtSWETT6* homolog was absent in *B. rapa* genome and *AtSWETT8*, *AtSWETT9*, *AtSWETT10*, as well as *AtSWETT13* have only one respective orthologous gene in the *B. rapa* genome. The expansion or loss of some *SWEET* genes was also found in potato (Manck-Götzenberger and Requena, 2016), tomato (Feng et al., 2015), rice (Yuan and Wang, 2013) and *B. napus* (Jian et al., 2016). The expansion of some *SWEET* family genes of the *Brassica* genome suggests their possible functional differentiations in response to the environmental conditions.

In order to better understand the role of *SWEET* gene family during colonization of *B. rapa* by *P. brassicae*, we firstly performed a gene identification in genomic level. The phylogenetic study revealed that the *BrSWEET* family genes were classified into 4 clades, an agreed feature defined in other higher plants as well (Kühn et al., 2010; Patil et al., 2015). Proteins from clade III were highly conserved among *Arabidopsis* and *Brassica* species, as shown by the strong support of branches separating the three protein groups, whereas in the other clades the identification of orthologous genes between species was more difficult. In this research, 32 *BrSWEET* genes, most of the closely related genes in family exhibit similar motif compositions, and these motifs were arranged in the same order in the C terminal of all BrSWEETs. It suggested that there are functional similarities in the SWEET family genes. However, the order of motif arranged in the N terminal was different. Motif 1-4/5-6-7 was found in clade I, II, and IV, but motif 7-2-3-5-6 in clade III. Previous studies of sugar transport properties showed that the *SWEET* genes in Clade I, II, and IV were monosaccharide (glucose, fructose, galactose, and so on) transporters, the Clade III *SWEET* genes were sucrose (disaccharide) transporter (Chen et al., 2010, 2012; Lin et al., 2014). So, we speculated that the different motifs of N terminal MtN3 domain between Clade I, II, IV, and Clade III result in the specificity of the *SWEET* genes in transporting different sugars. In other word, the variation of the structural motifs among BrSWEET family suggested functional diversity of the *SWEET* genes.

Most of the characterized or predicted *SWEET* genes encoded membrane proteins with 7 TM helices harboring 2 MtN3/saliva domains (Chen et al., 2010). In consistency with this, the majority of *SWEET* genes identified in *B. rapa* contained 7 TM helices. In spite of the variation of structural motifs, the two MtN3/saliva domains structured with TM1-3 and TM5-7 are conserved among BrSWEETs. However, protein sequences of *SWEET* genes with 4 (*BrSWEET7b*) or 6 (*BrSWEET2b*, -*4b*, -*8*, -*14c*, and -*17b*) TM helices distributed in the 2 MtN3/saliva domains were found. Interestingly, protein sequences of BrSWEET3b and BrSWEET15c with respective 3 and 4 TM helices were also found, which were previously only reported in bacteria and archaea (Wang et al., 2014). Expression profiling revealed that *BrSWEET* genes containing less than 7 TM helices were expressed in various tissues, indicating that they are possible functional genes although their substrate molecules and specific functions remains to be further studied.

### Expression Patterns of the Paralogous *BrSWEET* Genes in *B. rapa*

In this study, a total of 28 paralogous *BrSWEET* genes corresponding to 15 *AtSWEETs* were found in *B. rapa*. Expression profiling revealed that the paralogous genes of 6 *BrSWEET* (*BrSWEET1*, *-2*, *-11*, *-12*, *-13*, *-17*) gene families transcribed in all tested three tissues: roots, hypocotyls, and leaves, implying their functional redundancy and important role in vegetative growth stages. All clade III members of SWEETs were highly expressed in flowers, leaves and seeds, but not in roots in *B. napus* (Jian et al., 2016). In our result, all clade III BrSWEETs were transcribed in roots except BrSWEET9. Additionally, four genes (*BrSWEET4a*, -*14c*, -*15b*, and -*16a*) were also constantly expressed in all tissues, whereas their corresponding paralogs showed different expression patterns, indicating a possible gene function divergence which is commonly found in Brassica species (Chen et al., 2012; Jian et al., 2016). The remaining paralogous genes were either expressed in a tissue-specific manner or not expressed. For example, among 3 paralogous genes of *BrSWEET5*, *BrSWEET5a* and *5b* were not expressed in all tested tissues, except *BrSWEET5c* expressed in the hypocotyls. However, the induction of *BrSWEET5b* and *BrSWEET5c* were observed in the roots and hypocotyls of both CR and CS NILs after *P. brassicae* infection. Similarly, ortholog of SWEET5b in potato was also induced in response to fungal infection, but not expressed in control tissues (Manck-Götzenberger and Requena, 2016). *BrSWEET3b*, -*4b*, -*7a*, -*15c*, and -*16b* were also *P. brassicae*-inducible, while showed different expression profiling to their paralogs. Coincident with BrSWEET*3b* and -*7a*, induction for SWEET3b and SWEET7a orthologs in rice and potato was also observed in response to mycorrhiza colonizations (Fiorilli et al., 2015; Manck-Götzenberger and Requena, 2016). These findings further suggested that the different orthoglous *BrSWEET* genes may play the similar roles for sugar transport in response to the biotic interactions during plant evolutionary process.

### Involvement of the *BrSWEET* Genes in Clubroot Susceptibility

Several studies revealed that pathogens competed for sugars with infected host cells by hijacking host sugar efflux systems for disease development, being accompanied by highly induced expression of sugar transporter genes (Siemens et al., 2006; Doidy et al., 2012; Lemoine et al., 2013). *P. brassicae* also needs sugars from hosts for completing its life cycle, thereby inducing gall formation as an additional sink. Thus, regulation of those sugar transporters might determine the consequence of the plant-pathogen interactions.

To investigate the sugar redistribution during the *B. rapa*–*P. brassicae* interaction, we firstly detected the content of three soluble sugars in both leaves and roots of CS and CR NILs. In higher plants, sucrose is the main form of long distance transportation of carbohydrates from the source to the sink. In *A. thaliana*, it was found that sucrose was accumulated in uninfected leaves, but not in the leaves of *P. brassicae*-infected plants (Evans and Scholes, 1995), that is consisted with our results. Additionally, we also detected a decrease of sucrose together with an increase of glucose and fructose in clubroot galls in accordance with previous metabolic analysis in galls of both *A. thaliana* and Brassica species during development of clubroot disease (Brodmann et al., 2002; Mitchell and Rice, 2010), as well, contents of glucose and fructose in leaves of CS-NILs dramatically decreased. All these suggested that sucrose produced in *P. brassicae*-infected plants was exported from the leaves, probably rapidly metabolized, and further transferred into the clubroot pathogens.

Some *SWEET* genes showed no expression in normal growth/developmental conditions, but were transcriptionally induced by *P. brassicae* infection, including six genes (*BrSWEET3b*, *-4b*, *-5b*, *-5c*, *-8*, and -*9*) in the roots, seven genes (*BrSWEET3a*, -*3b*, -*4b*, *-5b*, *-7a*, -*8*, and -*16b*) in the hypocotyls, and three genes (*BrSWEET8*, -*9*, and *-15c*) in the leaves. Furthermore, inducted expression of several *BrSWEET* genes was at much higher levels in the CS-NIL than that of CR-NIL upon *P. brassicae* infection either in leaves (BrSWEET*2b*, -*14c*, -*15b*, -*15c*, -*16a*, -*17a*), hypocotyls (BrSWEET*9*, -*16a*) or roots (BrSWEET*1a*, -*2a*, -*9*, -*11a*, -*15c*). It is expected that these genes are responsible for transporting sugars from the leaf to the sink root tissues associated with *P. brassicae* colonization. Some other studies have demonstrated that increasing of *SWEET* expressions is to facilitate sugar transports to the non-plant sinks created by the phytopathogen infection or sugar secretions into the soil as carbon supply for pathogen growth (Patrick, 1989; Chen et al., 2010). Most of those genes belong to Clade III *SWEETs* which function as sucrose transporters responsible for long distance sugar transportation (Chen et al., 2010, 2012). Several studies have reported the involvement of Clade III members in various plant pathogenic systems, special SWEET11 orthologous genes in canola (*B. napus*) (Jian et al., 2016) and rice (Chen et al., 2010; Cernadas et al., 2014; Chandran, 2015). In our study, *Arabidopsis sweet 11* mutants showed significantly lower disease index value and the *P. brassicae* DNA content compared to that of wild-type plants after *P. brassicae* inoculation, revealing that the *BrSWEET11* played a crucial role during clubroot disease development. Hence, the enhanced sucrose transport from the leaf source tissues to the clubroot galls might be the main carbon transfer pathway in clubroot disease establishment, and the Clade III *SWEETs* play important roles in this progress. Whereas, notably some other *BrSWEET* genes were highly expressed in CR-NIL. Whether enhanced expressions of these genes contribute to host defense responses or are required to maintain carbon supplies for plant growth and development remains to be further investigated.

Competition for sugars at the plant–microbe interface could be controlled by the SWEET family transporters, and modulation of these transporter activities may determine the consequence (susceptibility or resistance) of the interaction (Toyofuku et al., 2000). However, this study did not examine the possible involvement of *SWEET* genes as potential susceptibility factors to the *P. brassicae* pathotypes. Presumably, *SWEET* genes of *P. brassicae* may also participate in competing for sugars in a manner of coevolving between host varieties and pathogen specializations (pathotypes), which is worthy of further study.

## Conclusion

We showed here that the infection caused by the virulence *P. brassicae* triggered the induction of Chinese cabbage SWEET transporters that could enhance sugar transport and modulate redistribution of sugar contents in host tissues during disease development. Pathogen-triggered alterations of sugar transport and redistributions would facilitate sugar acquisition by the pathogen from infected plant cells. Taken together, the susceptible *P. brassicae* infection triggered induction of specific *BrSWEET* members and increased cellular glucose and fructose contents specifically in the roots of the CS-NIL, implying that successful *P. brassicae* may manipulate long distance translocation of host sugars from the site where the sugars are synthesized to the site where the clubroot pathogen colonizes and reproduces, probably through regulating a specific set of the host *BrSWEET* genes. However, sugar transports in higher plants are very complex. Other sugar transporters are not only required for plant growth, development and reproduction, but also involved in plant–pathogen interactions (Chu et al., 2006; Yuan et al., 2011; Chandran, 2015; Liu et al., 2016). Whether the homologs of these sugar transporters play roles during the interactions between Chinese cabbage and *P. brassicae* remains to be further investigated.

## Author Contributions

HL performed the experiments, analyzed the data, and drafted the manuscript. XL participated in the data analysis and wrote the manuscript. YX and JJ helped to draft the manuscript. YW contributed analysis tools and helped to draft the manuscript. ZP conceived the study, participated in its coordination, and helped to draft the manuscript. All authors have read and approved the final manuscript.

## Conflict of Interest Statement

The authors declare that the research was conducted in the absence of any commercial or financial relationships that could be construed as a potential conflict of interest.

## Footnotes

1 http://brassicadb.org

2 http://www.expasy.org/tools/

3 http://gsds.cbi.pku.edu.cn

4 http://www.cbs.dtu.dk/services/TMHMM-2.0/

## Supplementary Material

FIGURE S1 Predicted domain structures of the 32 BrSWEETs. Blue lines indicate regions with cytosolic locations, pink lines indicate regions with apoplastic localization, and red boxes indicate putative transmembrane domains.

FIGURE S2 Amino acid sequence alignment of 32 BrSWEET proteins. Multiple sequence alignment for 32 BrSWEET protein sequences was conducted using the ClustalX program. Amino acid (AA) sequences with black color indicate their 100% identity among the homologous proteins, AA sequences with blue color indicate their homology between 75 and 100%, and AA sequences with pink color indicate their homology between 50 and 75%.

FIGURE S3 Distribution of conserved motifs of BrSWEETs. The phylogenetic tree was built using the unrooted tree that was generated in MEGA5 using the neighbor-joining (NJ) method. Motif analysis was performed using MEME 4.0 software as described in the methods. The phylogenetic tree was shown in left panel, and motif sizes are indicated at the bottom of the figure. Different motifs are indicated by different colors numbered from 1 to 10. The same number in different proteins refers to the same motif.

FIGURE S4 Expression patterns of the *SWEET* genes in the hypocotyl of Chinese cabbage after infection of *P. brassicae*. Hierarchical clustering and heatmap representation show time course responses of *SWEET* gene expressions in the hypocotyl of Chinese cabbage after *P. brassicae* infection. The expression levels of genes are presented using fold-change values transformed to Log_2_ format. The data obtained by quantitative RT-PCR correspond to the levels of *SWEETs* in total RNA samples extracted from roots before and after infection of *P. brassicae*. The data indicate the relative expression levels normalized to that of the internal control *Actin* or *18srRNA*. Gray color means no signal detected, while red and green colors correspond to up- and down-regulations of the *SWEET* genes, respectively.

TABLE S1 Information of *B. rapa SWEET* genes.

TABLE S2 qRT-PCR primer sequences.

TABLE S3 Raw data of disease index (DI) value and relative pathogen DNA quantities detected between *Arabidopsis* wild type and *sweet11* mutants.
