# Supplementary figures and images for: Genome Wide Identification and Expression Profiling of SWEET Genes Family Reveals Its Role During Plasmodiophora brassicae-Induced Formation of Clubroot in Brassica rapa
Source: Front Plant Sci. 2018 Feb 28;9:207. doi: 10.3389/fpls.2018.00207 (PMC5836591; doi:10.3389/fpls.2018.00207)

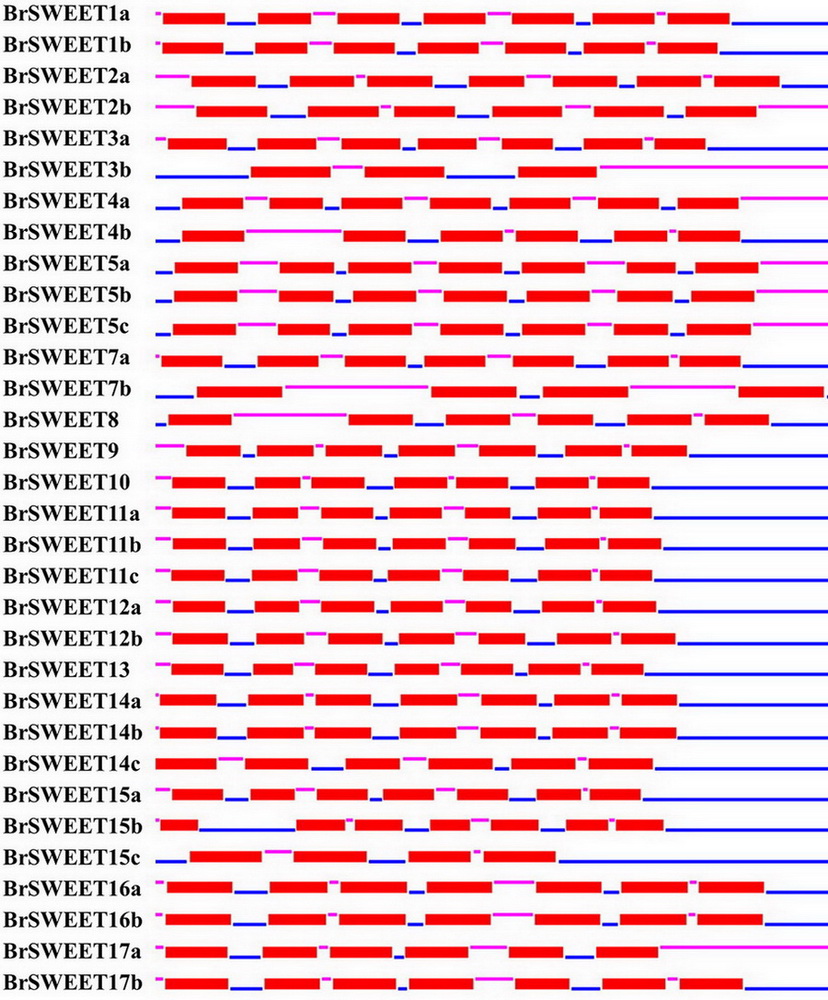

Supplement: FIGURE S1 — Predicted domain structures of the 32 BrSWEETs. Blue lines indicate regions with cytosolic locations, pink lines indicate regions with apoplastic localization, and red boxes indicate putative transmembrane domains. [file Image_1.JPEG]

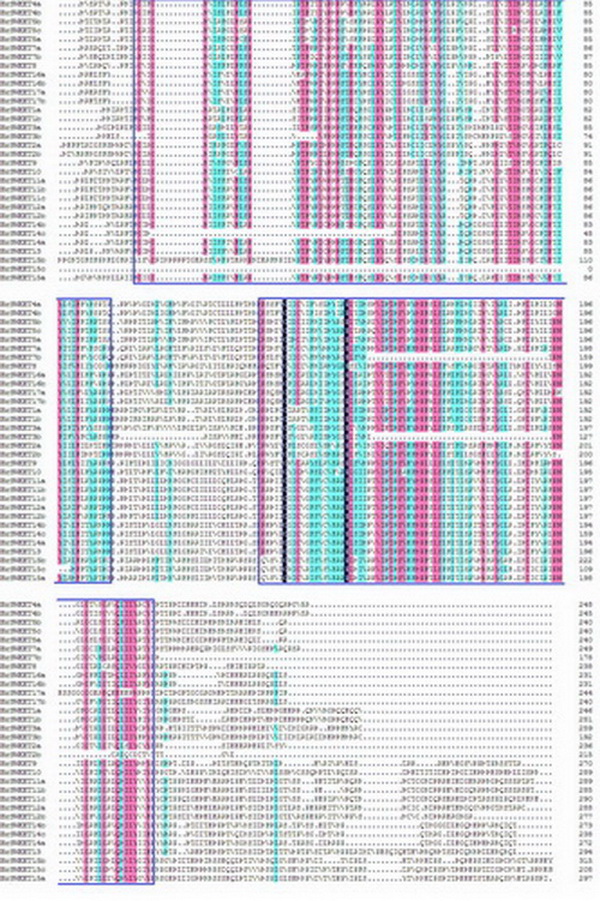

Supplement: FIGURE S2 — Amino acid sequence alignment of 32 BrSWEET proteins. Multiple sequence alignment for 32 BrSWEET protein sequences was conducted using the ClustalX program. Amino acid (AA) sequences with black color indicate their 100% identity among the homologous proteins, AA sequences with blue color indicate their homology between 75 and 100%, and AA sequences with pink color indicate their homology between 50 and 75%. [file Image_2.JPEG]

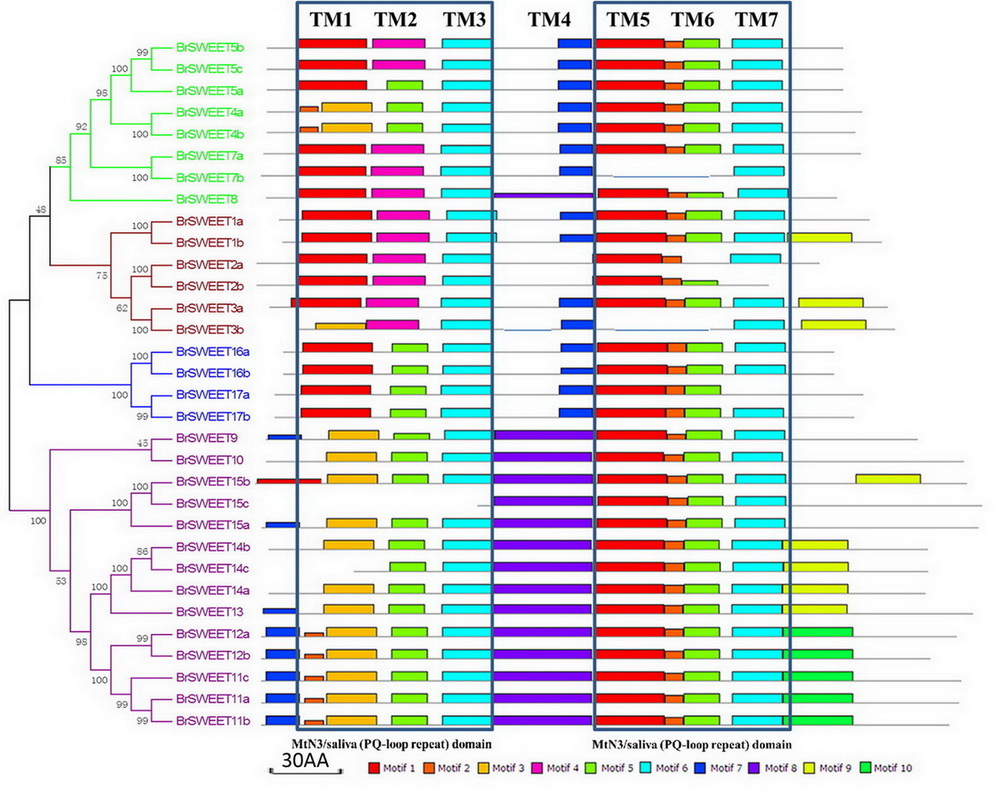

Supplement: FIGURE S3 — Distribution of conserved motifs of BrSWEETs. The phylogenetic tree was built using the unrooted tree that was generated in MEGA5 using the neighbor-joining (NJ) method. Motif analysis was performed using MEME 4.0 software as described in the methods. The phylogenetic tree was shown in left panel, and motif sizes are indicated at the bottom of the figure. Different motifs are indicated by different colors numbered from 1 to 10. The same number in different proteins refers to the same motif. [file Image_3.JPEG]

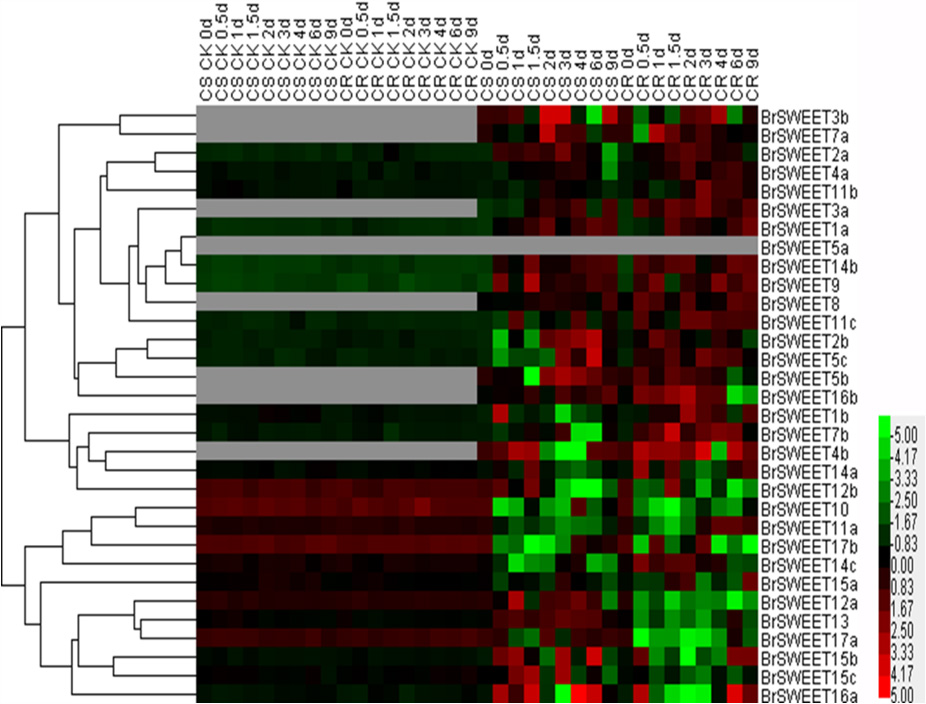

Supplement: FIGURE S4 — Expression patterns of the SWEET genes in the hypocotyl of Chinese cabbage after infection of P. brassicae. Hierarchical clustering and heatmap representation show time course responses of SWEET gene expressions in the hypocotyl of Chinese cabbage after P. brassicae infection. The expression levels of genes are presented using fold-change values transformed to Log2 format. The data obtained by quantitative RT-PCR correspond to the levels of SWEETs in total RNA samples extracted from roots before and after infection of P. brassicae. The data indicate the relative expression levels normalized to that of the internal control Actin or 18srRNA. Gray color means no signal detected, while red and green colors correspond to up- and down-regulations of the SWEET genes, respectively. [file Image_4.JPEG]
