# Supplementary material for: Genome Wide Identification and Expression Profiling of SWEET Genes Family Reveals Its Role During Plasmodiophora brassicae-Induced Formation of Clubroot in Brassica rapa
Source: Front Plant Sci. 2018 Feb 28;9:207. doi: 10.3389/fpls.2018.00207 (PMC5836591; doi:10.3389/fpls.2018.00207)
Supplement: TABLE S1 — Information of B. rapa SWEET genes. [file Table_1.DOCX]

**Table S1. Information of *B. rapa* *SWEET* genes**

| **Gene name** | **Gene accession No.** | **Chromosomal location** | **Location** | **Exon** | **TMD** | **MtN3/saliva (PQ-loop repeat) domain position** | **Theoretical pI/Mw** | **Gene length** | **AA length** | **E-value** | **Arabidopsis homologous** |
| --- | --- | --- | --- | --- | --- | --- | --- | --- | --- | --- | --- |
| BrSWEET1a | Bra017916 | A06 | 8531013..8532244 | 6 | 7 | 8-94,131-213 | 9.30 / 27185.23 | 1232 | 246 | e-127 | AT1G21460 |
| BrSWEET1b | Bra016421 | A08 | 17617578..17618868 | 6 | 7 | 8-94,131-213 | 9.19 / 28837.01 | 1291 | 251 | e-126 | AT1G21460 |
| BrSWEET2a | Bra021577 | A01 | 23576930..23578595 | 6 | 7 | 18-104,138-221 | 7.59 / 26567.48 | 1666 | 236 | e-107 | AT3G14770 |
| BrSWEET2b | Bra027314 | A05 | 21002455..21003761 | 6 | 6 | 18-104,138-207 | 9.16 / 24077.84 | 1307 | 215 | 9e-91 | AT3G14770 |
| BrSWEET3a | Bra022636 | A02 | 7369617..7371074 | 6 | 7 | 8-97,133-219 | 8.71 / 29327.76 | 1458 | 259 | e-105 | AT5G53190 |
| BrSWEET3b | Bra003075 | A10 | 5464198..5465848 | 4 | 3 | 6-86 | 7.64 / 21593.34 | 1651 | 192 | 2e-69 | AT5G53190 |
| BrSWEET4a | Bra025317 | A06 | 21145739..21147433 | 6 | 7 | 12-98,132-215 | 9.07 / 27488.74 | 1695 | 248 | e-122 | AT3G28007 |
| BrSWEET4b | Bra033034 | A02 | 22295276..22297037 | 6 | 6 | 12-98,133-215 | 8.94 / 27416.71 | 1762 | 245 | e-119 | AT3G28007 |
| BrSWEET5a | Bra035879 | A09 | 3753202..3754740 | 6 | 7 | 11-94,132-218 | 9.04 / 27300.77 | 1539 | 240 | e-113 | AT5G62850 |
| BrSWEET5b | Bra029245 | A02 | 25812459..25813833 | 6 | 7 | 11-94,132-218 | 8.15 / 26971.38 | 1375 | 240 | e-108 | AT5G62850 |
| BrSWEET5c | Bra029244 | A02 | 25820140..25821571 | 6 | 7 | 10-94,132-218 | 8.14 / 26826.03 | 1432 | 240 | e-98 | AT5G62850 |
| BrSWEET7a | Bra035270 | A09 | 16467275..16468808 | 5 | 7 | 10-99,136-220 | 9.62 / 27362.11 | 1534 | 249 | e-112 | AT4G10850 |
| BrSWEET7b | Bra000725 | A03 | 12702139..12703812 | 4 | 4 | 11-100,137-176 | 8.63 / 19580.56 | 1674 | 178 | 5e-74 | AT4G10850 |
| BrSWEET8 | Bra025595 | A04 | 7977927..7979212 | 6 | 6 | 9-98,135-218 | 8.97 / 26793.78 | 1286 | 238 | 6e-87 | AT5G40260 |
| BrSWEET9 | Bra000116 | A03 | 9354182..9355694 | 6 | 7 | 11-98,132-218 | 9.18 / 30125.02 | 1513 | 270 | e-114 | AT2G39060 |
| BrSWEET10 | Bra022761 | A03 | 6890031..6891355 | 6 | 7 | 11-96,131-215 | 9.29 / 33041.99 | 1325 | 289 | e-125 | AT5G50790 |
| BrSWEET11a | Bra018039 | A06 | 9429836..9431906 | 6 | 7 | 12-99,133-218 | 9.18 / 32068.06 | 2071 | 289 | e-151 | AT3G48740 |
| BrSWEET11b | Bra019560 | A06 | 13263479..13265878 | 6 | 7 | 12-99,133-218 | 9.32 / 31584.52 | 2400 | 285 | e-149 | AT3G48740 |
| BrSWEET11c | Bra029914 | A01 | 14292131..14294908 | 6 | 7 | 12-99,133-218 | 9.04 / 32343.51 | 2778 | 290 | e-143 | AT3G48740 |
| BrSWEET12a | Bra009700 | A06 | 17244307..17246021 | 6 | 7 | 12-99,133-218 | 9.07 / 31781.95 | 1715 | 288 | e-144 | AT5G23660 |
| BrSWEET12b | Bra026487 | A09 | 3209585..3211449 | 6 | 7 | 12-99,133-218 | 9.13 / 30494.73 | 1865 | 277 | e-141 | AT5G23660 |
| BrSWEET13 | Bra040489 | Scaffold000211 | 86998..89197 | 6 | 7 | 10-97,132-217 | 9.05 / 32747.84 | 2200 | 294 | e-143 | AT5G50800 |
| BrSWEET14a | Bra010477 | A08 | 13951936..13953577 | 6 | 7 | 8-96,130-216 | 9.18 / 29916.69 | 1642 | 272 | e-131 | AT4G25010 |
| BrSWEET14b | Bra013854 | A01 | 8084759..8086390 | 6 | 7 | 8-96,130-216 | 9.29 / 30105.98 | 1632 | 273 | e-128 | AT4G25010 |
| BrSWEET14c | Bra019197 | A03 | 25772583..25774202 | 5 | 6 | 12-61,95-181 | 9.08 / 26408.58 | 1620 | 238 | e-108 | AT4G25010 |
| BrSWEET15a | Bra023394 | A02 | 2136659..2138430 | 6 | 7 | 13-99,134-218 | 8.26 / 33156.31 | 1772 | 297 | e-130 | AT5G13170 |
| BrSWEET15b | Bra006185 | A03 | 2393566..2401305 | 6 | 7 | 55-123,158-242 | 8.86 / 35814.01 | 7740 | 316 | e-116 | AT5G13170 |
| BrSWEET15c | Bra008850 | A10 | 12940345..12941235 | 4 | 4 | 45-129 | 7.74 / 23314.58 | 891 | 209 | 7e-86 | AT5G13170 |
| BrSWEET16a | Bra021190 | A01 | 22618948..22620602 | 6 | 7 | 6-93,131-203 | 8.69 / 25754.61 | 1655 | 231 | e-113 | AT3G16690 |
| BrSWEET16b | Bra001638 | A03 | 17645985..17647579 | 6 | 7 | 6-93,131-203 | 9.06 / 25679.67 | 1595 | 231 | e-108 | AT3G16690 |
| BrSWEET17a | Bra012752 | A03 | 22453768..22456653 | 6 | 7 | 6-92,128-213 | 9.01 / 27311.85 | 2886 | 240 | e-121 | AT4G15920 |
| BrSWEET17b | Bra038060 | A08 | 6881574..6899808 | 6 | 6 | 6-92,128-188 | 8.76 / 26444.05 | 18235 | 244 | 6e-95 | AT4G15920 |

Gene accession No., chromosomal location, coding sequence location, gene nucleotide numbers, and protein length (AA) were deduced from the Chinese cabbage (*B. rapa*) sequence database (<http://brassicadb.org>). The number of exons (Exon) was predicted by Gene Structure Display Server (GSDS) (<http://gsds.cbi.pku.edu.cn>). The number of transmembrane domains (TMD) was predicted by TMHMM Server v2.0. MtN3/saliva (PQ-loop repeat) domain position was predicted by Batch Web CD-Search Tool ([http://www.ncbi.nlm.nih.gov](http://www.ncbi.nlm.nih.govbrassicadb.org)). Theoretical pI/MW of each *BrSWEET* protein were calculated using the compute pI/Mw tool of ExPASy (<http://www.expasy.org/tools/>).
