# Supplementary material for: Genome Wide Identification and Expression Profiling of SWEET Genes Family Reveals Its Role During Plasmodiophora brassicae-Induced Formation of Clubroot in Brassica rapa
Source: Front Plant Sci. 2018 Feb 28;9:207. doi: 10.3389/fpls.2018.00207 (PMC5836591; doi:10.3389/fpls.2018.00207)
Supplement: TABLE S2 — qRT-PCR primer sequences. [file Table_2.DOCX]

**Table S2 qRT-PCR primer sequences**

| Genes | Forward primers | Reverse primers |
| --- | --- | --- |
| Actin | 5’-TATGCTCTTCCACATGCTATTC-’3 | 5’-CCTTACGATTTCACGCTCTG-’3 |
| 18SrRNA | 5’-GTTCTTAGTTGGTGGAGCGATTT-’3 | 5’-ACCTGTTATTGCCTCAAACTTCC-’3 |
| BrSWEET1a | 5’-CTCTCGTCTCTCTCTTTGCCCTC-’3 | 5’-TTCGTCTTTATCACCAACCTCAT-’3 |
| BrSWEET1b | 5’-TCTACGTCCTGATCTTCCTTTTC-’3 | 5’-ACTCTTCGTCTTTACCACCAACC-’3 |
| BrSWEET2a | 5’-TGCCTTTGGTATGGCTCACCTTAC-’3 | 5’-AGCATCCTCATCTTGGTTTTCTCG-’3 |
| BrSWEET2b | 5’-GTGACTGTGAACTCCGTTGG-’3 | 5’-TCGCTGTTGAATAACCCGTA-’3 |
| BrSWEET3a | 5’-TTTTCATTTCTGGCTAGCTCCCT-’3 | 5’-TCCCATTTGCTCATCACTGTTGT-’3 |
| BrSWEET3b | 5’-AAAGTGATAGAGACAAAAAG-’3 | 5’-AATAAAACAAAGAAAGGGAG-’3 |
| BrSWEET4a | 5’-TCATTTGGGTTATTTATGCCCTT-’3 | 5’-ACTCCTTTAGCTTGTCCCTGGTT-’3 |
| BrSWEET4b | 5’-ATTTGGGTTATTTATGCCCTTATT-’3 | 5’-CTTCATCTTTTGGTGTTGTCTTGT-’3 |
| BrSWEET5a | 5’-ACCAACGATGATAAAGATATGGAAGA-’3 | 5’-GTAGCGAAGATGAAGAAGATAGTGAC-’3 |
| BrSWEET5b | 5’-GATCTATGCTTGTTGGGATCTTAT-’3 | 5’-ACTTCACGCTCTTTGTCTTTATTA-’3 |
| BrSWEET5c | 5’-CCTCGTCATCACCAATAATG-’3 | 5’-TGGCTAAAGACAGGGAGAAC-’3 |
| BrSWEET7a | 5’-GAAAATGGTAATAAGAACGAAAAGC-’3 | 5’-TGAAAAAGAAAGAAATACTCACAGC-’3 |
| BrSWEET7b | 5’-CTCTACAACACACTACCAAAGAACG-’3 | 5’-ACAAAATGGGGAAATAATTAACAAG-’3 |
| BrSWEET8 | 5’-TCCTTCTTTCTCTTCTTCTCGCC-’3 | 5’-CATACAGTTCATCATCGTTGCCA-’3 |
| BrSWEET9 | 5’-GTTTGTGCTGCTTACAGTCTCG-’3 | 5’-CTTTGTCCTTATCACCTTCCTC-’3 |
| BrSWEET10 | 5’-AGAAGTGCAGAGTTCATGCCGT-’3 | 5’-TGGTTCCAAAACCTTTGTCCCA-’3 |
| BrSWEET11a | 5’-GCTCCTTTATGGTCTTGCTCTCAA-’3 | 5’-GCAGCTTCCACTTCTTTCTCTTCC-’3 |
| BrSWEET11b | 5’-CGTATGGCTCCTTTATGGTCTTGC-’3 | 5’-CTTAGCGGCTTCCACTTCTTTCTC-’3 |
| BrSWEET11c | 5’-CCTCCTCGTCACCATCAACTC-’3 | 5’-AATCCGACACATATTCCTCCG-’3 |
| BrSWEET12a | 5’-CACACGTGCAAAGATAATCGGAG-’3 | 5’-AGAGCAAGACCATAAAGAAGCCA-’3 |
| BrSWEET12b | 5’-TGCTTTGGCTTTACTACGCTACA-’3 | 5’-CGACACAGATTCCTCCGATGATC-’3 |
| BrSWEET13 | 5’-CCATTGTCCTTGTCTGCGAG-’3 | 5’-ACTGGAGTGGGCATAACGGT-’3 |
| BrSWEET14a | 5’-TAGTGCGATGCTATGGATTTACT-’3 | 5’-TGATATTCTTGCTTTCTTGTTGG-’3 |
| BrSWEET14b | 5’-TCCTCTTCATCACCTACGCTAACAA-’3 | 5’-GAGAAAAGGGCATAAACTCCACACT-’3 |
| BrSWEET14c | 5’-TTTGCCGCTATTATTCTTGTCTGT-’3 | 5’-TTTTTACCTTTCACGTGTTAAACG-’3 |
| BrSWEET15a | 5’-TTCACGCTTTCTTTCTTCCTCACT-’3 | 5’-AAAACCATTTGTACCAACCCTAGT-’3 |
| BrSWEET15b | 5’-CACCCTCTCTTTCTTCCTCACCAT-’3 | 5’-TTCCGACACACCTAACTGACTCAT-’3 |
| BrSWEET15c | 5’-TCGTGATGAGTCCGTTAGGTGTGT-’3 | 5’-AGTTGCCGGCTCCTTCTCTTTAGT-’3 |
| BrSWEET16a | 5’-GGTTCTCCTCTTTCCGCTATTG-’3 | 5’-TGTTTCTTTGTGGTGGTTGATT-’3 |
| BrSWEET16b | 5’-TTGGAAGATAGTGAAACGGAGAT-’3 | 5’-CAAAGAAGAGGAAAATGAGAACG-’3 |
| BrSWEET17a | 5’-AGCGAAAATCGACGGAGGACTAC-’3 | 5’-GGGGCATAAATGAGAAAAAGGGA-’3 |
| BrSWEET17b | 5’-TCCCTTTTTGTCTTATACGCACC-’3 | 5’-CTCATCTTTAAACGCACTTCTCG-’3 |
